# Man and His Enemies

**Published:** 1889-05-04

**Authors:** 


### The Doctor's Pulpit.

### MAN AND HIS ENEMIES.

### Barriers against Marriage.

"Cursed be the social wants that sin against the strength of youth."

Thus sang Tennyson in the vigour of his manhood. He aimed a hard blow at things as they then were, but with small evident result, The blow, however, was well worth aiming, and the state of mind which gave impulse to the flexors and extensors of the arm is still more deserving of practical consideration. What made the polished and dignified Tennyson stir the witch's broth and give such dramatic utterance to a deep-mouthed imprecation on the ways and manners of the times that then were? Something, one feels certain, that had bitten, like a keen acid, deeply into his own life's experience. Mere speculative philosophers and prize-poem contributors do not give origin and direction to explosions of that kind. Who was the "shallow-hearted" Cousin Amy? Had her poet-relative loved her with all the swelling and resistless force of a first *grande passion?* And had he wanted to marry her when his means were all too limited? And had Amy first returned the passion in her feeble way, and then received a better offer and taken it? And had the pride and wounded self-love of the poet been so intolerably humiliated that nothing would mollify and ease the wound but a bellowing cannon of rhyme and a dramatic display of malediction?

Whatever was the origin of "Locksley Hall," few of us are so old as to have outlived our sympathy with the noble ardour of the poet's love, and with the natural and not ignoble anger which flamed out upon the woman's feeble unworthiness. The social wants that stood in the way of the poet's happiness were solid barriers, which even the consuming energy of youthful passion could not burn down. In the case of Tennyson, though they were enemies at the time, his transcendant genius turned them into friends. Such social wants still bar the way to happiness for many a youthful pair; but instead of proving friends in disguise they are enemies, which often mark the starting point of ruin.

If a man should write a pamphlet on "Latter day Difficulties," the difficulty which he would have to place at the head of his list would be the difficulty of getting married. It is much easier for the average man to undertake an expedition to China, or a journey round the world on a bicycle, than to set up house with a wife. Marriage, according to the old theory, should be the union of two loving hearts; it too often is in practice the union of two calculating heads. The "socials wants" which were so reat nearly half a century ago, when "Locksley Hall" first fired true lover's hearts, are now more numerous, more varied, and more immeasurable than Tennyson ever dreamed of. For the average young man and woman to fall in love is about the most Quixotic piece of folly of which the inexperienced simpleton can be guilty. The only wise course for many a man is not to dream of love until he is forty; and if a girl wants to marry before she is five-and-twenty, she must almost certainly make up her mind to marry a widower or a bachelor of at least forty-five. The rent, and the taxes, and the food, and the clothing, and the summer holiday, and the social courtesies, and the servants, and the washing-bills and a thousand other "ands" which might be added, are simply overwhelming in actual experience, however shadowy and vaporous they may appear to young eyes that look at them through the spectacles of love.

In considering a subject of this kind the mind, in its absolute hopelessness of better things, takes refuge in a kind of cynical amusement. But the business is not one which should excite a smile. The unmarried men between twenty-four and forty—what becomes of them? Where do they spend their time, their money, their strength? Are they anchorites? They will not marry and face the stern struggle of limited means. No! But many of them, solely because they cannot marry, deliberately throw over a pure and simple morality, and give themselves up body and mind to that kind of physical self-indulgence which they think they can best afford. Is it necessary to ask what the consequences are, or to set them forth in detail? It would be impossible to set them forth. If any man had the genius and the courage to paint the life of the multitudes of young men who are deterred from marriage by the impassable mountains of social wants, no other man or woman would believe that the world could possibly be the scene of so much baseness and bestial cruelty on the one hand, or of so much physical misery and despairing remorse on the other. Tragedy, ruin, despair, are as common in great cities as the smoke of the morning chimneys, or the lighting of the street lamps at the close of day.

It would be an interesting enquiry to enter upon, if it could by any means be carried out, how many and what class of men feel themselves impelled to seek a remedy for this condition of things? Who are they who see most clearly the ruins of so many human temples? Are they the scientific evolutionists, or the experimental physiologists, or the practising physicians? Or are they none of these classes, but the out-of-date theologians of the Catholic Church, the Protestant Church, the Established faith, and the Salvation Army? It is a question of the supremest practical significance. No one can deny the facts of the physical and mental ruin of thousands of men in early and mid-life every year. All the wars in the world do not match in destructiveness the devouring jaws of the monster of self-indulgence, which is the creature of a too costly civilisation. Where are the good Samaritans who hasten to the relief of the wounded man, and who can by no means be content until they have carried him to a place of safety? Does any cynical sceptic smile at the question? Let him be answered out of his own mouth. What is the object of your physical researches, of your special and general hospitals? Is it not the cure and removal of many of the very evils which result from the condition of things we have been describing; and if it be a good thing to cure such evils, would it not be a ten thousand times better thing to prevent them, and to nip all their terrible consequences in the bud?

One of two things is plainly demanded in the interests of the young men upon whom the largest share of the world's thinking and working will so speedily fall. Either marriage should be made easier, or their virtue should be made stronger. Either the environment should be improved, or the resisting fibre of the mind and body, which are surrounded by its destructive influences, should be toughened. Is either of these things possible? Are they both possible? Undoubtedly they are both possible. It is our constant contention in these pages that human brains united with and impelled by human conscience can accomplish anything which lies within the bounds of possibility. That the physical and moral environment of man can be improved is amply demonstrated by the fact that it has been constantly and steadily improving for at least two thousand years. That man's virtue and resisting power can be strengthened is equally clear, and for a similar reason. On any hypothesis this is so. The theory of the evolutionist admits and demands it as decisively as that of the theologian. What the world wants is not wrangling and speculation, either on one side or the other, but clearness of vision, practicality of aim, and the steady energy of goodwill which resolves that, whatever others do, it at least will spend its strength in building and repairing the fabric of the man and of the commonwealth, and not in pulling them down. The most powerful antiseptic and tonic mankind has ever seen is inward, intelligent virtue. The body and the soul physiscians who really desire to arm the young man against all possible enemies, should labour with well-directed purpose to strengthen and increase that.

